# Induction of pre-hospital emergency anaesthesia i-PHEA: a national survey of UK HEMS practice

**DOI:** 10.1186/s12873-023-00897-5

**Published:** 2023-10-31

**Authors:** Mark Hodkinson, Kurtis Poole

**Affiliations:** Thames Valley Air Ambulance, Stokenchurch House, Oxford Road, Stokenchurch, HP14 3SX UK

**Keywords:** Pre-hospital emergency anaesthesia, Rapid sequence induction, Pre-hospital emergency medicine, Induction, Anaesthesia, Helicopter emergency medical service.

## Abstract

**Background:**

Pre-hospital emergency anaesthesia is a critical intervention undertaken by helicopter emergency medical teams. Previous studies informed current practice for induction regimes, using a standardized approach of fentanyl, ketamine and rocuronium. There may be a trend towards post-induction hypotension attributed to the induction regime used. Several new combinations of fentanyl, ketamine and rocuronium are emerging in clinical practice. There is currently no consensus on what induction regimes should be used.

**Methods:**

A semi-structured survey was distributed to the medical leads of all UK air ambulance organisations between December 2022 and February 2023. Responses that were returned within the study period were included. Exclusions included missing data, declined participation and failure to return the survey within the data collection period. The survey sought to establish provision of pre-hospital emergency anaesthesia and current induction regimes for stable, unstable and post-cardiac arrest patients. Data was extracted from Microsoft Forms into Excel. Descriptive statistics were used to analyse survey response rate, provision of PHEA and induction regimes. The survey was endorsed by the National HEMS Research and Audit Forum.

**Results:**

19 air ambulance organisations responded (response rate 86%). The majority of organisations provide over 100 pre-hospital emergency anaesthetics per annum (79%, n = 15/19). A standard combination of fentanyl, ketamine and rocuronium is used as a primary induction regime in haemodynamically stable patients by 52% of services (n = 10/19). In haemodynamically compromised patients, fentanyl was omitted or pracititioner choice emphasized by 79% of services (n = 15/19). There was variability in the dose of rocuronium from 1 mg/kg to 2 mg/kg throughout services.

**Conclusion:**

There is variability in the approach to pre-hospital emergency anaesthesia. There is a growing dataset that would enable development of a registry to better understand induction regimes and the impact on patient physiology. Organisations are increasingly adopting a patient centered, practitioner choice model towards induction of anaesthesia.

**Supplementary Information:**

The online version contains supplementary material available at 10.1186/s12873-023-00897-5.

## Background

There are 22 air ambulance organisations operating helicopter emergency medical services (HEMS) in the United Kingdom, with the majority operating solely through charitable donations. HEMS organisations in the UK support various NHS ambulance services to deliver pre-hospital emergency medicine and critical care.

Pre-hospital emergency anaesthesia (PHEA) is one of the most critical interventions undertaken by HEMS teams. The National Institute for Health and Care Excellence (NICE) quality standard for airway management advocates early management of the airway and ventilation, within 45-minnutes from the point of injury, which includes the provision of PHEA at the scene in systems where this can be achieved [1]. There has broadly been a standardized approach to the induction of PHEA in trauma patients, utilizing fentanyl, ketamine and rocuronium in dose regimes for stable and unstable patients [2]. There may be a trend of post-induction hypotension associated with this approach as the availability and scrutiny of data increases [3, 4]. In the context of trauma, a single episode of hypotension is associated with increased mortality, particularly in head injured patients [5, 6]. Post-induction hypotension may be associated with drug regimens or patient factors. This may contribute to alternative PHEA regimes amongst critical care teams [3].

Several new iterations of the fentanyl, ketamine and rocuronium combination are emerging across the UK [4]. These alternative regimes may provide comparable induction of anaesthesia and favourable intubating conditions whilst reducing the incidence of post induction hypotension. Further exploration around UK delivery of PHEA and any variability is warranted.

To date, limited studies [7] have sought to establish the provision of PHEA across the UK nor the induction regimes used. This service evaluation aims to establish the incidence of PHEA in UK HEMS and compare the induction regimes used to establish current UK practice.

## Method

A semi-structured survey developed for this service evaluation (see supplementary material), using Microsoft Forms, was distributed to the medical leads at all (22) UK air ambulance organizations via the National HEMS Research and Audit Forum (NHRAF). Data collection occurred over a 3-month period, from December 2022 to February 2023. Reminders to complete the survey were sent each month to maximise survey response.

The survey contained a series of structured questions to establish the area of service, level of PHEA provision and the primary regimes used in stable, unstable and post return of spontaneous circulation (ROSC) patients. Content was informed by relevant literature [2–4]. The survey was designed to establish current clinical practice within the last 24-month period.

### Inclusion criteria

- UK HEMS organisations providing charity or Government funded air ambulance operations.
- Completed & returned survey within the service evaluation period.

### Exclusion criteria

- Missing data.
- Declined participation.
- Survey not returned within service evaluation period.

### Data analysis

Data was extracted from the Microsoft Form into an Excel spreadsheet. Organisational names were removed from the data to create an anonymised sample. Descriptive statistics were used to describe the overall response rate, volume of PHEA provision and induction regimes for stable, unstable and post-ROSC patients. Due to the small volume of free text comments, these were grouped together into common themes by the first author.

### Ethics & service evaluation approval

In accordance with national legislation, this project was not considered research by the NHS Health Research Authority decision tool. This service evaluation is not considered to be research and formal ethical approval was not required [8].

Endorsement to conduct this service evaluation was sought from the National HEMS Research and Audit Forum (NHRAF). The service evaluation was also registered with the Thames Valley Air Ambulance internal Audit, Improvement, and Research (AIR) group, who maintained oversight of the service evaluation.

#### Informed consent

was gained from the participant responding on behalf of the organisation during the survey.

## Results

19 air ambulance organisations responded to the survey, a response rate of 86% (n = 19/22). There were no duplicate responses or exclusions. Table 1 shows a breakdown of demographics and induction regimes used. Tables 2 and 3 summarise the variations on the standard 3:2:1 and 1:1:1 induction regimes used.

**Table 1:** Main results

	n	%
**Service operational hours**		
12 h (day only)	2	11
19 h (day & late)	6	32
24 h	8	42
Other*	2	11
Not reported	1	5
**PHEA provision (average number of PHEA in last 12 months)**		
> 100	15	79
< 90	1	5
< 60	1	5
< 30	1	5
Not reported	1	5
**Primary induction regime - haemodynamically stable patient**		
Standard 3:2:1 regime	5	26
Variation of standard 3:2:1 regime (summarised in Table 2)	5	26
Ketamine & rocuronium only	4	21
Practitioner choice	5	26
**Primary induction regime - haemodynamically compromised patient**		
Standard 1:1:1regime	3	16
Fentanyl 1mcg/kg + ketamine 1 mg/kg + rocuronium 2 mg/kg	1	5
Ketamine 1 mg/kg + rocuronium 1 mg/kg	10	53
Practitioner choice	5	26
**Primary induction regime - post ROSC patient**		
Fentanyl, ketamine & rocuronium	1	5
Fentanyl, midazolam & rocuronium	5	26
Ketamine & rocuronium	4	21
Practitioner choice	9	47
**Primary regime for maintenance of anaesthesia**		
Ketamine infusion	3	16
Propofol infusion	6	32
Bolus dosing (including ketamine, midazolam, fentanyl, morphine)	7	37
Practitioner choice	3	16
*mix of 12, 19 and 24 h across 7 day period		

**Table 2:** Variation of standard 3:2:1 regime

	n
**Variation of standard 3:2:1 regime (haemodynamically stable patient)**	
Fentanyl 3mcg/kg + ketamine 2 mg/kg + rocuronium 2 mg/kg	1
Fentanyl 2mcg/kg + ketamine 2 mg/kg + rocuronium 1 mg/kg	2
Fentanyl 1mcg/kg + ketamine 2 mg/kg + rocuronium 1 mg/kg	1
Fentanyl 1mcg/kg + ketamine 2 mg/kg + rocuronium 2 mg/kg	1

The overwhelming theme arising from free-text comments was the flexibility for individual clinician choice of anaesthetic induction agent and dose regime, that consider individual patient requirements as opposed to fixed agents and dose regimes (Table 3). The second most common theme was the increased dose of rocuronium, varying from 1.4 mg/kg to 2 mg/kg.

**Table 3:** Common themes from free-text comments

*“We prefer our clinicians to adapt their PHEA to each patient rather than have fixed stable or unstable regimens which don't cover every eventuality”*
*“Clinicians at our organisation are allowed to use their own clinical judgement rather than sticking to a specific ratio for a clinical scenario”*
*“We support the team modifying regimes to support individualised patient care. Our most commonly performed anaesthetic involves fentanyl / ketamine / rocuronium”*
*“We have recently changed our drug regimes and do not subscribe to didactic dose regimes. We use fentanyl, ketamine, rocuronium for trauma patients and allow the clinician to judge dose dependent on age, GCS and stability”*

## Discussion

It is believed that this is the first survey to date that has sought to establish PHEA provision and what, if any, are the common induction regimes used in UK HEMS practice. Most services can be considered high volume PHEA providers [7], with 79% (n = 15/19) delivering over 100 anaesthetics in the previous 12-months. This gives the potential for a large data set by which the induction regimes and response to induction of anaesthesia can be monitored in detail and consider the effects on patient physiology and outcome. Future work should focus on the development of a national HEMS PHEA registry that would further inform clinical practice.

Two studies conducted by the Pre-hospital Trainee Operated Research Network (PHOTON) sought to establish PHEA provision across UK HEMS in 2020, as part of wider studies into geo-temporal provision of PHEA and compliance against the NICE 45-minute quality standard [9, 10]. Of note, the geo-temporal analysis of PHEA only reported provision at two points in time; a Tuesday and a Sunday in the summer of 2018 [10]. Whilst this study goes some way to describing the provision of PHEA across a 24-hour period, it is difficult to generalize the findings across a 365-day period, where several factors may affect the provision and subsequent delivery of PHEA, including availability of staff, weather issues that may affect flight, and variances in regional populations. Data presented confirms that the UK HEMS systems deliver an increasing number of PHEAs per annum (n = 1755 PHEAs in 12-month period, 1 April 2017 to 31 March 2018 inclusive [9]), which are similar to the results found in this survey (15 providers delivering over 100 PHEA per annum). Neither study explicitly examined induction regimes utilized.

Except for post-ROSC patients, there is consensus amongst all responding organisations in the approach to what agents are used in the induction of anaesthesia, namely a combination of fentanyl, ketamine and rocuronium. Rocuronium appears to be the only paralytic agent that is used in UK HEMS practice, representing a change in practice since a 2017 survey by Burgess et al [7] where several other agents were available. A limitation of this study was the pre-loaded questions that focused solely on the use of rocuronium and did not consider any other paralytic agent. However, there were no additional paralytic agents highlighted in free-text responses, suggesting that rocuronium is the only agent in use. Analysis of free-text responses indicated a move towards increased dosing of rocuronium across all patient groups, ranging from 1.4 mg/kg to 2 mg/kg. This represents a further change in practice from the dose regimes described by Lyon et al [2] in 2015. The optimal dose of rocuronium remains unclear [11], but there is a trend toward improved first pass intubation success where higher doses (> 1.4 mg/kg) are administered as part of the induction (92.2% first pass success (dose > 1.4 mg/kg) versus 88.4% first pass success (dose < 1.0 mg/kg)) [11]. Organisations responsible for PHEA delivery should consider moving toward a higher dose strategy of rocuronium, and further work is warranted to inform this change.

There are several plausible explanations for favorable intubating conditions with higher dose rocuronium, particularly when used in combination with video laryngoscopy. The higher dose rocuronium may saturate the synapse more quickly, and the increased availability of the drug in a patient with poor cardiac output increases drug distribution [11]. Modern, plastic based video laryngoscopes are less stimulatory than the colder, metal direct laryngoscopes, and as such reduces the stimulatory effects when inserted into the airway. However, further work is required to better understand the association of dose and intubating conditions.

As of 2019, a consensus statement from the European HEMS and Air Ambulance Committee Medical Working Group specified no particular drugs or regimes for PHEA, in favour of a regime that included an induction agent, an opioid and a fast acting muscle relaxant [12]. UK practice has favoured fentanyl, ketamine and rocuronium as either a 3:2:1 or 1:1:1 regime since 2015 [2]. This represented a shift from the use of agents such as etomidate and suxamethonium [13], and the findings of this survey suggest that there has been a further change in how induction of anaesthesia is delivered, as evidenced by the limited consensus approach to drug regimes in hemodynamically stable patients (Table 1). There is a trend to moving away from the standardized 3:2:1 approach, to a modified approach that is becoming increasingly practitioner choice and dependent upon the patient’s physiology, injuries, age, and comorbid state (Table 2). Although fentanyl remains commonplace in pre-hospital anaesthetic practice, there widespread variability in its use, and the dose used, varying from 1mcg/kg to 3mcg/kg in stable patients. This service evaluation did not seek to establish any cause or effect of the use of fentanyl, and further in-depth exploration of the effects on patient’s physiology is warranted. However, when considering hemodynamically compromised patients, there is a clear signal that most organisations utilize an induction regime of ketamine and rocuronium only (53%, n = 10/19). The development of a national PHEA registry would add further evidence to the role of fentanyl in PHEA, and organisations should continue to adopt a pragmatic, patient centered approach to anaesthesia.

The European Resuscitation Council post-resuscitation care guidelines do not recommend one agent over another for post-ROSC anaesthesia, in favour of a combination of a sedative, analgesic and rapid onset neuromuscular blocking agent [14]. Ketamine may cause an increase in heart rate and blood pressure, and increased myocardial oxygen demand, though it is unclear whether this adversely affects clinical outcome. Although increased heart rate and blood pressure may be favourable in the bradycardic, hypotensive post ROSC patient, increased myocardial oxygen demand may worsen ongoing ischemia. The use of fentanyl and midazolam therefore seem a reasonable alternative to ketamine in post-ROSC anaesthesia. Despite this, a quarter of organisations surveyed (26%, n = 5/19) adopt a regime that uses ketamine during post-ROSC anaesthesia, with 47% (n = 9/19) organisations using a variable, practitioner choice approach to anaesthesia. The emphasis, as in all pre-hospital anaesthetics, is to balance haemodynamic instability during induction with adequate anaesthesia. Acknowledging differences in patient physiology should be a key consideration when selecting drugs and doses.

There are important safety considerations when using a standardized approach to PHEA, including reduced cognitive loading in often stressful situations, and reduced drug errors [13]. The trend in moving away from didactic regimes highlight the maturity of UK HEMS practice and the increase in delivery of PHEA. Robust governance systems that monitor practice are commonplace in UK HEMS, and there is growing recognition that not all patients will fit within a prescribed regime.

### Limitations

Duplication of survey response was minimised by specifically targeting organisational medical leads. This may introduce bias towards what is written in policy and procedure compared to actual clinical practice. Clinical practice and delivery of anaesthetic drugs may vary considerably between operators, and it is then difficult to quantify and measure each individual variance.

## Conclusion

This study has highlighted the variability in anaesthetic practice across UK HEMS, and the shifting trend toward practitioner choice over didactic dose regimes that may not be suitable for all patients. Whilst this adds to the evidence around PHEA, a dedicated PHEA registry would enable close monitoring of induction regimes, the physiological response and overall patient outcomes. Organisations are increasingly adopting a patient centered, practitioner choice model towards induction of anaesthesia. Further exploration of the initial dose of rocuronium of 1.4-2.0 mg/kg is warranted.

### Electronic supplementary material

Below is the link to the electronic supplementary material.

Supplementary Material 1

## Data Availability

The datasets generated and analysed during the current study are available from the corresponding author on reasonable request.

## Abbreviations

PHEA: Pre-hospital emergency anaesthesia
HEMS: Helicopter emergency medical service
UK: United Kingdom of Great Britain and Northern Ireland
NHRAF: National HEMS Research and Audit Forum
ROSC: Return of spontaneous circulation
AIR: Audit, improvement, and research group
GCS: Glasgow Coma Scale
PHOTON: Pre-hospital Trainee Operated Research Network
