# Supplementary material for: Induction of pre-hospital emergency anaesthesia i-PHEA: a national survey of UK HEMS practice
Source: BMC Emerg Med. 2023 Oct 31;23:126. doi: 10.1186/s12873-023-00897-5 (PMC10617087; doi:10.1186/s12873-023-00897-5)
Supplement: Supplementary file 1 — Supplementary Material 1 [file 12873_2023_897_MOESM1_ESM.docx]

**INDUCTION OF PRE-HOSPITAL EMERGENCY ANAESTHESIA**

**i-PHEA: a national survey of UK HEMS practice**

**SUPPLEMENTARY MATERIAL**

**Survey questions**

| **Number** | **Question** | **Response options** |
| --- | --- | --- |
| 1 | Please identify your service | Drop down list of UK air ambulance charities |
| 2 | How many hours does your service currency operate? | 12 hours (day only)  19 hours (day & late)  24 hours  Other (state) |
| 3 | Does your service provide pre-hospital emergency anaesthesia? | Yes  No  Other (state) |
| 4 | On average, how many pre-hospital emergency anaesthetics did your service perform in the last 12-months? | 0-10  11-20  21-30  31-40  41-50  51-60  61-70  71-80  81-90  91-100  >100  Other (state) |
| 5 | What is your PRIMARY induction regime for haemodynamically stable patients? | Fentanyl 3mcg/kg + ketamine 2mg/kg + rocuronium 1mg/kg  Fentanyl 2mcg/kg + ketamine 2mg/kg + rocuronium 1mg/kg  Ketamine 2mg/kg + rocuronium 1mg/kg  Other (state) |
| 6 | What is your PRIMARY induction regime for haemodynamically compromised patients? | Fentanyl 1mcg/kg + ketamine 1mg/kg + rocuronium 1mg/kg  Ketamine 1mg/kg + rocuronium 1mg/kg  Other (state) |
| 7 | What is your PRIMARY induction regime for post-ROSC patients? | Fentanyl 3mcg/kg + ketamine 2mg/kg + rocuronium 1mg/kg  Fentanyl 1mcg/kg + ketamine 1mg/kg + rocuronium 1mg/kg  Fentanyl 1mcg/kg + midazolam 0.05-0.1mg/kg + rocuronium 1mg/kg  Other (state) |
| 8 | What is your PRIMARY regime for maintaining anaesthesia? | Ketamine bolus  Ketamine infusion  Fentanyl bolus  Midazolam bolus  Propofol infusion  Other (state) |
| 9 | Please add any other comments/information on pre-hospital emergency anaesthesia | Free text comment |
